# Experimentally induced animal models for cognitive dysfunction and Alzheimer's disease

**DOI:** 10.1016/j.mex.2022.101933

**Published:** 2022-11-24

**Authors:** Deepthi Rapaka, Paul C. Adiukwu, Veera Raghavulu Bitra

**Affiliations:** a A.U. College of Pharmaceutical Sciences, Andhra University, India; b School of Pharmacy, University of Botswana, Gaborone, Botswana

**Keywords:** Alzheimer's disease, Animal models, Induction dose, Validation

## Abstract

Alzheimer's disease (AD) is a progressive neurodegenerative disorder characterised pathologically by the presence of extracellular amyloid plaques and the intracellular neurofibrillary tangles, along with inflammation, and a compromised antioxidant system. Significant insights into the neurobiology to better understand the pathophysiology of AD and to evaluate the possibility of cutting-edge therapy strategies, can be obtained through the selection of a well-designed experimental animal model. From the transgenic to chemical/drug-induced models, none of them represents the complete picture of Alzheimer pathology and incidence of cognitive dysfunction. Researchers did not explain why one model was preferred over another, did not consider how the pathological phenomena were formed (spontaneously, experimentally, or by genetic manipulation), and did not address the traits of the species that affect the results. There is a lack of concordance between preclinical models and clinical trials that could be due to variety of reasons such as incomplete models, choice of animal species, lack of variability, and the validity of the models. To provide greater translation of preclinical AD studies to clinical trials proper designing of the model is essential. This review provides a brief recap ranging from animal doses to their induction mechanism and common limitations of the chemical-induced AD models.

• Animal models may fail to replicate the exact pathology of the disease

• Validity of the model is essential for proper translation of pathology from animal models to human disease

• Appropriate induction doses need to be administered.

## Graphical abstract

**Figure:**
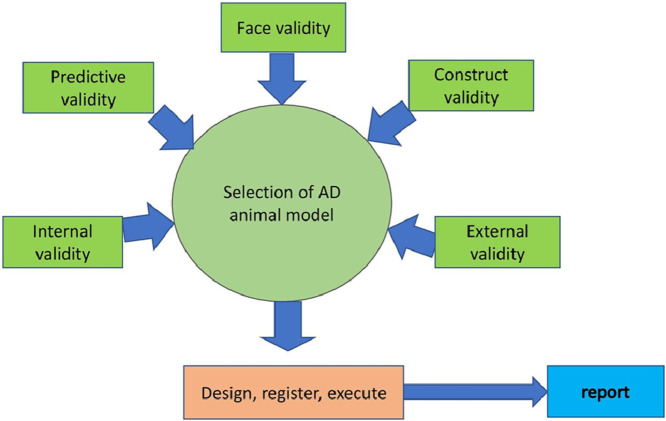

Specifications table

**Table:** 

Subject area	
More specific subject area	*Alzheimer's disease*
Name of the reviewed methodology	*Induction of Alzheimer's disease in animal models*
Keywords	*Induction; Animal models; chemical-induced.*
Resource availability	*pubmed /Elsevier/ Nature/ Wiley / Springer /Medknow*
Review question	*Animal models of Alzheimer's disease*
Name & Reference of original method	*N.A.*

## Method details

### Introduction

Alzheimer's disease (AD) is the most common form of dementia. The prevalence increases logarithmically with age. According to the latest statistics, the prevalence of AD will raise to 13.8 million from 5.8 million in Americans by 2060. 121,499 deaths were reported in the year 2019 [1]. The clinical manifestations include memory impairment, language, visuospatial, behavioural perceptions, and disturbances [2]. AD pathogenesis can be evident earlier before the symptoms become deceptive [3].

Experimental animal models are essential for further understanding of AD pathology. Most animal models currently being used are chemical/drug-induced models. A well-designed experimental model is required not only to understand the underlying disease mechanisms but also to produce a novel therapeutic effect. It is exaggerating to know that over the past 19 years, nearly 400 clinical trials have failed (unsuccessful) in the synthesis of a new drug for AD. Data obtained from animal studies was not found to be properly translated into clinical studies [4]. Humans share extensive genetic and physiologic homology with mammals such as rodents, and where the likeness of a disease state can be demonstrated, knowledge gained by the study of the model may inform interpretation of human disease conditions. It is challenging to understand how this might be applied to successful translation of preclinical research in Alzheimer's disease, especially in cognitive and memory dysfunction, a condition whose phenomenology surpasses that which could be observed in animals. The animal models are categorised based on the disease characteristics, whether they are acquired by induction (chemical/drug/ physical), spontaneous (naturally occurring), genetic changes (transgenic models) [5]. In this study, we discussed the chemical/drug-induced models for Alzheimer's disease (Table 1).

**Table 1:** Animal models for Alzheimer's disease.

S.No	Model	Species, age, gender	Biomarkers	Dosage regimens	References
1.	Streptozotocin induced AD.	• Male, Wistar albino rats aged 3-4months • Male/female Sprague-Dawley rats aged 2-5months • Male/female Cynomolgus monkey aged 5-8 years	Amyloid plaques, tauopathy, gliosis, atrophy of parenchyma, Neurochemical alterations, oxidative stress, neuroinflammation	3mg/kg (i.c.v) at 48hr intervals in two divided doses for rodents 2mg/kg (i.c.v) divided into four dosages for monkeys	[6], [7], [8], [9]
2.	Aβ-induced AD	• Male Fisher rats aged 18-20 weeks. • Male, Long-Evans hooded aged rats 2-3months. • Male/female, aged Rhesus monkeys aged 25-28 years, • Male/female aged Marmoset monkeys 8-10years. • Male/female aged vervet monkeys aged 19-23 years.	Amyloid plaques, Tau phosphorylation, cholinergic deficits, neuroinflammation, glutaminergic alterations	Acute/cont infusion of Aβ into 3^rd^ ventricle (5µg/1µl) Continuous infusion (10/20 µg/3days) into right ventricle	[10], [11], [12], [13], [14]
3.	Scopolamine- induced- AD	• Male albino rats aged 4-months. • Male/ female Swiss albino mice aged 10-12 weeks	Tau pathology, Cholinergic impairment, monoamine alterations, neuroinflammation	1, 3mg/kg (i.p.) 0.3-0.5mg/kg (i.p.) 72µg/0.5µl (i.c.v)	[16], [17], [18]
4.	AF64A induced memory deficits	• Male Wistar-Imamichi rats, aged 7-8weeks. • Sprague-Dawley rats aged 5 weeks, male	Cholinergic deficits, astrogliosis	2nmol/2µl intra cerebrally 8nmol bilaterally into striatum 6nmol/6µl i.c.v	[19,20]
5.	Alcohol- induced memory deficits	• Male, Wistar rats aged 2-3 months • Male Sprague-Dawley rats aged 2 months	Deficits in spatial memory, oxidative stress Neuroinflammation, neuronal loss.	(20% w/v, 4 g/kg, p.o. for 28 days)	[21], [22], [23], [24], [25], [26]
6.	Cholesterol and copper sulfate model	• Male New Zealand white rabbits aged 3-4months.	Amyloid-plaques, oxidative stress, gliosis, apoptosis.	2% cholestrol+0.12ppm of copper ions	[27], [28]
7.	Colchicine model of AD.	• Albino rats aged 6-8weeks, male	Amyloid plaques, neuroinflammation, cognitive deficits, NMDA activation, neurodegeneration.	(7.5 µg/10 µl,icv)	[29], [30], [31]
8.	Immunotoxin ^192^IgG-saporin model	• Male Sprague-Dawley rats aged 2-months • Male Wistar rats aged 8-12 weeks	Cholinergic deficits, cognitive deficits, synaptic changes, glial changes.	^192^IgG-saporin 4 µg/0.3 µl, i.c.v., 375ng/0.3 µl i.c.v.,	[32], [33], [34]
9.	Lysophosphatidic acid induced AD	• Mouse neuroblastoma N2a cell line	Amyloid production, upregulates BACE1 expression, cognitive changes, oxidative stress.	25µM	[35,36]
10.	Metal (aluminium) model	• Male Wistar rats aged 6-12 months. • Male Sprague-Dawley rats aged 6-16weeks.	Amyloid plaques, Tangles, neurotransmitter alterations, cognitive deficits, oxidative stress, gliosis, neuroinflammation.	Al (5mg/kg, 10mg/kg, i.p, 60days) Al (4.2mg/kg, i.p, 28 days) Al (100mg/kg, oral)	[37], [38], [39], [40], [41], [42]
11.	Methionine	• Male Wistar albino rats aged 3 months	Cholinergic deficits, endothelial dysfunction, neuroinflammation, gliosis, cognitive deficits, oxidative stress	1.7g/kg per day p.o for 32 days 1.7 g/kg per day p.o. for 8 weeks	[43,44]
12.	Okadaic (OKA) acid	• Male/female Sprague-Dawley rats aged 2-3months	Tau phosphorylation, Amyloid β plaques, oxidative stress, cognitive deficits.	Okadaic acid (70 ng/day icv, for 14 days)	[45,46]
13.	(Excito)toxin	• Male Sprague-Dawley rats aged 2-3 months	Oxidative stress, apoptosis, cognitive deficits, excitotoxicity,	Ibotenic acid (10 µg/5 µl; icv.,)	[47,48]
14.	Clonidine-induced memory deficits	• Male Wistar rats aged 2-3 months	Cognitive deficits	0.1mg/kg, i.p	[49], [50], [51]
15.	Clozapine-induced memory deficits	• Female Sprague-Dawley rats aged 2-3 months	Cognitive deficits	1.25mg/kg, s.c; 2.5mg/kg s.c.,	[55,56]
16.	Lignocaine-induced memory deficits	• Male Wistar rats aged 3months.	Cognitive deficits	2% lidocaine in 1µl, i.c.v	[52], [53], [54]
17.	Cycloheximide-induced memory deficits	• Male naïve Sprague-Dawley rats	Cholinergic deficits, cognitive disturbances, neurotransmitter alterations	1mg/kg; 150mg/kg; s.c.,	[57,58]
18.	Phenytoin induced memory deficits	• Male Wistar rats aged 2-3months	Oxidative stress, cognitive deficits	75mg/kg, i.p.,	[59], [60], [61]
19.	D-Galactose induced cognitive impairment	• Male C57L/B6 mice aged 6-8weeks • Male Swiss albino mice aged 9 weeks	Amyloid-β, neuroinflammation, oxidative stress, cognitive deficits.	d-gal (150 mg/kg) for 6 weeks.	[62], [63], [64], [65], [66], [67], [68]
20	Dizocilpine (MK-801) AD	• Male Wistar rats aged 8-10 weeks • Male C57L/B6 mice aged 7-9 weeks	Cognitive deficits, motor disturbances	0.15mg/kg,i.p.,	[69,70]
21.	Diazepam	• Male Swiss albino mice -young (3-4 months) and aged (12-15 months)	Cognitive deficits	1mg/kg., i.p	[15,75]

### Animal models

The following models are a few commonly used models and have been proven to offer better efficacy in the development of new therapeutics for the development of AD-like models.

***1. Streptozotocin-induced model of AD****:* Streptozotocin (STZ) is a glucosamine nitrosourea compound that belongs to the alkylating agents of anti-cancer drugs. This is the most widely employed model for induction of AD as it very closely resembles human sporadic AD. STZ at a sub-diabetogenic dose of 3mg/kg in two divided doses viz., i.c.v. route produces memory dysfunction like AD [7], [8], [9]. STZ infusion leads to enhanced oxidative and nitrosative stress, brain atrophy, neuronal loss, neuroinflammation, along with Aβ accumulation and tau hyperphosphorylation. Impaired synthesis of acetyl coenzyme A, ATP and creatine phosphate were observed. Increased activity of the acetylcholinesterase (AChE) enzyme along with apoptosis was reported with STZ administration. [6]

### Advantages

It is a reliable model for the studies on Alzheimer's disease.

It replicates the major pathological hallmarks such as the expression of amyloid plaques, neurofibrillary tangles, elevated oxidative stress, neuroinflammation.

It resembles the pathology of human sporadic AD.

It is a valid model for studying the pathology of AD.

### Limitations of the model

This is an invasive model that requires large number of animals because of its high mortality rate.

***2. Amyloid-β model of Alzheimer's disease****:* Amyloid-β plaques are the major hallmarks of this disease. Administration of the Aβ peptide produces cognitive dysfunction, followed by neurodegeneration. Intracerebroventricular administration of Aβ into the 3^rd^ ventricle for 14days leads to the accumulation of amyloid proteins and plaques in different regions of the brain [11], [12], [13], [14], particularly in regions of memory such as the hippocampus and cortex, followed by the amygdala and striatum [10]. This model produces behavioural changes apart from the alterations in biochemicals and neurochemicals, so it is proved to be more suitable for induction of AD [11], [12], [13], [14].

### Advantages

It replicates the pathological hallmarks such as the amyloid plaques, neurofibrillary tangles, and neurodegeneration.

Behavioral alterations can be evaluated.

This is a specific model for screening of AD drugs.

### Limitations of the model

Potential confounding effects of the invasive intracerebral injection.

***3. Scopolamine induced AD model:*** The cholinergic system plays a vital role in the pathogenesis of AD. Reduced cholinergic activity leads to cognitive deficits [15]. Scopolamine, a known anticholinergic agent, blocks the cholinergic muscarinic receptor and leads to over release of acetylcholine which eventually damages the hippocampus, cortex, and nucleus basalis. Scopolamine can be administered via both i.p and i.c.v. routes. This drug disrupts the cholinergic tracts in areas responsible for cognition and memory [16], [17], [18].

### Advantages

Provides ease of scopolamine administration.

Cholinergic deficits can be studied broadly using this model.

### Limitations of the model

The model fails to replicate the pathological hallmarks of the AD.

***4. (Ethyl choline mustard aziridinium ion) AF64A-induced AD model:*** AF64A is a neurotoxic product derived from choline. This chemical causes long-term damage to cholinergic neurons. Intracerebroventricular administration of AF64A produces cholinergic deficiency by altering acetylcholine levels and choline acetyltransferase activities in the cerebral cortex, hippocampus, and striatum [19,20], eventually leading to behavioural impairment along with neurochemical and biochemical changes.

### Advantages

The model is advantageous to study the cognitive deficits involved in learning and memory.

### Limitations of the model

Intracerebral injections could be difficult and may cause trauma in some animals.

The model fails to reproduce the hallmarks of the disease.

***5. Alcohol-induced memory deficits:*** Increased and elevated doses of ethanol have been known to cause memory deficits by affecting the encoding, storage, and consolidation [25]. Ethanol is known to impair hippocampal dependent learning and memory, eventually leading to the cholinergic dysfunction [25]. Intraperitoneal administration of ethanol dose dependently (0.25, 0.5, 1g/kg) impaired memory retention in rodent models [21], [22], [23], [24],26].

### Advantages

Does not require any invasive process for the induction of memory deficits.

Memory enhancing agents and the nootropic activity of the drugs can be evaluated.

It exhibits behavioural alterations similar to AD.

Cognitive deficits can be explored.

### Limitations of the model

The slow and time-consuming process.

The pathological hallmarks of AD were not replicated in this model.

***6. Cholesterol and copper sulfate model:*** Administration of cholesterol supplemented with copper sulfate in distilled water pathologically revealed the presence of senile plaques. The efflux of excess Aβ caused by cholesterol from the brain is attenuated by copper ions, which is the most likely mechanism for copper induced Aβ aggregation [27,28]. **Advantages**

Easy reliable and reproducible model.

The model is beneficial for exploring the fibrillar Aβ pathology.

### Limitations

The pathological hallmarks such as the presence of neurofibrillary tangles, neurodegeneration, were not replicated.

**7. *Colchicine model of AD:*** Colchicine is being used in the treatment of inflammatory disorders such as gout for decades. Colchicine causes neuronal cell death by binding to tubulin, the main structural protein of the microtubule, and causing microtubular depolymerization and destabilization. This is followed by the blockage of axonal transit and mitosis [29].

Administration of colchicine (i.c.v), a causes significant cognitive dysfunction in rodents [29], by disrupting the cholinergic tracts. It also causes changes in the other monoaminergic neurotransmitters such as depletion in dopamine, serotonin and nor-epinephrine in the hippocampus, caudate nucleus, and cerebral cortex [30,31]. The neurotoxicity is mediated through oxidative stress, and excitotoxicity via NMDA activation [29], [30], [31].

### Advantages

Replicates the behavioral, biochemical, and neurochemical alterations which are the main features of sporadic dementia of Alzheimer's type (SDAT).

Possible to screen nootropic agents and other cholinesterase inhibitors for memory.

NMDA activity and excitotoxicity can be explored.

### Limitations of the model

The model may require large number of animals due to high mortality rate.

Adverse effects associated with the colchicine such as (myoclonic twitches, aggressive behaviour, acoustic startle behaviour and reduced threshold to pain) may affect/ alter the study and may lead to more suffering of the animals, so ethical considerations are the key issues in this model.

***8. Immunotoxin Ig-saporin model:*** Neurons of the septum and diagonal band of Broca are the major source of cholinergic innervation in the hippocampus. Hippocampus is most effected region of the brain in AD, which is majorly associated with the learning and memory. Intracerebroventricular administration of ^192^IgG-saporin (Ig-saporin), induces degeneration of neurons and results in cholinergic deficits in various regions of the hippocampus [32,33]. This immunotoxin alters behavioral patterns in different behavioural tasks and impairs memory retention in animal models [34].

### Advantages

Behavioural and electrophysiological consequences of degeneration of cholinergic neurons can be evaluated.

Insights into hippocampal alterations are possible with this model.

Neurotoxicity and underlying mechanisms can be explored.

Cholinergic deficits can be broadly evaluated.

### Limitations

Invasive route of administration.

All pathological hallmarks were not expressed in this model.

**9. *Lysophosphatidic acid model:*** Growth cone collapse and neurite retraction are two effects of the bioactive phospholipid lysophosphatidic acid on neuronal cells [35,36]. Tau and other microtubule-associated proteins (MAPs) have higher levels of phosphorylation rate as a result of alterations caused by LPA in tubulin pools. Glycogen synthase kinase-3 is activated in response to LPA, which causes tau hyperphosphorylation. It happens in a variety of neuronal cells from many species in conjunction with the neurite retraction process [36]. It has been shown that LPA-induced neurite retraction in differentiated SY-SH5Y human neuroblastoma cells increases site-specific tau phosphorylation similar to that seen in Alzheimer's disease.

### Advantages

Tau phosphorylation, tauopathies and the related mechanisms could be explored.

### Limitations

Other pathological hallmarks were not expressed.

Limited to cell cultures.

***10. AlCl_3_-induced AD model****:* Aluminium (Al) acts as a neurotoxin and cholino toxin, Al can gain easy access into the body and the chronic exposure induces oxidative stress and damages the CA fields of hippocampus, apart from the structural modifications of the nicotinic receptors leading to memory loss [37,42]. It induces oxidative stress, alters the blood-brain barrier, leading to the apoptotic death of hippocampal neurons. It also promotes the expression of the amyloid [41], tau [38] proteins and alters the acetylcholine and other monoamine levels [40]. In previous studies, it has also been shown to alter the behavioural aspects of rodents. Finally, leading to the expression of all the pathological hallmarks of Alzheimer's disease [37], [38], [39], [40], [41], [42].

### Advantages

All the pathological hallmarks were replicated in this model.

Ease of aluminium administration.

This is an old, reliable, and reproducible model for the study of AD.

Less mortality rates.

### Limitations

The amyloid plaques expressed in this model were pathologically different from the senile plaques of human AD.

***11. Methionine model of AD:*** Treatment with L-methionine dramatically increases serum homocysteine levels and elevated homocysteine levels have been linked to oxidative stress and changes in the structure and function of cerebral blood vessels, both of which are important contributors to cerebral vascular dysfunction. It is known that oxidative stress and vascular dysfunction play a significant role in the pathology of AD and vascular dementias [43,44]. Memory acquisition and retrieval are significantly impaired in AD due to structural abnormalities in the cerebral capillaries, which also cause neuronal malfunction and death [44] Additionally, it has been demonstrated that hyperhomocysteinemia is neurotoxic [43]. This neurotoxicity is due to an excess of N-methyl-D-aspartate receptors, elevated oxidative stress, increased cholinesterase activity, tau phosphorylation and the toxicity caused by amyloid-β peptides [44].

### Advantages

Beneficial for studying the vascular dementia.

Easy and safer route of administration of methionine.

Useful for screening of drugs with nootropic activity.

### Limitations

Does not replicate the pathological hallmarks of AD.

***12. Okadaic acid (OKA) model:*** OKA is a potent protein phosphate inhibitor (PPI/2A). Administration of OKA via the intracerebroventricular route for 14 days leads to cognitive dysfunction and significant pathological alterations and elevated oxidative stress. It causes the hyperphosphorylation of tau by expressing neurofibrillary tangles and also decreases the phospho-GSK3β [45,46].

### Advantages

Beneficial for screening of dementia drugs.

All the pathological hallmarks were expressed.

### Limitations

Administration of OKA could be difficult due to the invasive procedure, so there are chances of high mortality.

***13. Toxin model of AD:*** Ibotenic acid is an excitotoxin and has been shown to cause damage to the nucleus basalis magnocellularis (NBM). Based on the finding that AD patients exhibit degenerative changes in the nucleus basalis of Mynert (nbM), the human counterpart of the NBM, lesions of the NBM have been proposed as an experimental model for the disease. Additionally, similar to what has been observed in AD patients, the NBM lesioned animal exhibits decrease in cholinergic markers, such as acetylcholine levels, release and turnover of acetylcholine, choline uptake, choline acetyl transferase and acetylcholinesterase activity, and number of muscarinic cholinergic receptors, in the frontal cortex [47,48].

### Advantages

The cholinergic scenario in patients with AD matches with the pathology in rodent models hence, it is considered as a valid model for studying memory deficits in AD.

### Limitations of the model

High mortality rate and invasive procedure.

***14. Clonidine -induced memory deficits:*** Clonidine, a presynaptic adrenergic agonist, has become well established as a useful and relatively safe therapeutic agent. Clonidine has been described to inhibit the bioelectrical activity of noradrenergic neurons of the locus coeruleus, via activation of α2 adrenoceptors. clonidine also induces synchronization in cortical EEG pattern in rodents, cats, and rabbits [51]. Clonidine at doses of 0.1–1 mg/kg increases the firing of 5-HT cells in the dorsal raphe nucleus, facilitating the adrenergic influence on the 5-HT neurons, which suggest clonidine induced serotonergic transmission underlies the amnesic effect of the drug [50].

### Advantages

The underlying mechanisms and the influence of monoamines can be evaluated.

Behavioural effects can be studied using this model.

Cognitive deficits can be evaluated.

### Limitations

The model does not replicate the AD hallmarks.

The model is non-reliable.

The underlying mechanisms cause the depressive effects of clonidine such as inhibition of locomotor and exploratory activities, suppression of avoidance behaviour and suppression of self-stimulation in laboratory animals.

***15. Lignocaine-induced memory deficits***: Lignocaine, a local anaesthetic commonly employed for the arrythmias is known to block the voltage-gated sodium channels. Lidocaine injected bilaterally into the rodent brain, disrupted spatial information consolidation [54] and short-term and long-term memory [52] by disrupting the CA1 pyramidal cell function [52,53]. It is also known to impair memory processes by disrupting granule cell output functions [51].

### Advantages

Long-term and short-term memory dysfunctions can be explored using this model.

### Limitations

The model does not replicate the pathological hallmarks and not reliable AD model.

The route of administration is invasive, so there are chances of mortality.

The drug is known to have potential adverse effects such as drowsiness, mental clouding, dysphoria, muscle twitching, fall in blood pressure.

***16. Clozapine-induced memory deficits:*** Behavioral changes are common in dementia, particularly in its later stages. Agitation, hostility, paranoid delusions, hallucinations, sleep disturbances, including nocturnal wandering, incontinence, and (stereotypical) vocalizations are the most prevalent disorders. These symptoms are often overlooked and treated with anti-psychotics, such as clozapine. Experimental evidence indicated working memory and attention impairment in rodents. Even though, the underlying mechanism appears to be unknown. However, the studies indicated that loss of nicotinic cholinergic β2 receptors are associated with the cognitive deficits [55,56].

### Advantages

The underlying mechanisms and the influence of cholinergic system can be evaluated.

Behavioural effects can be studied using this model.

Only cognitive deficits can be evaluated.

### Limitations

The model does not replicate the pathological hallmarks and not reliable AD model.

The underlying mechanisms cause the other adverse effects associated with the use of clozapine such as excessive drowsiness, vision problems, voluntary movement changes and the blood parameters have to be closely monitored even low doses are administered.

***17. Cycloheximide-induced memory deficits:*** Cycloheximide, a potent protein synthesis inhibitor that interferes with translocation, and impairs development of long-term memory [57]. It is known to induce memory consolidation deficits majorly via disturbances in the cholinergic and GABAergic systems and increased serotonergic activity [57,58]. Administration of cycloheximide in rodents significantly impaired memory consolidation in the behavioural tasks [58].

### Advantages

This model is beneficial to study the cognitive deficits associated, and the role of various neurotransmitters in memory deficits.

Ease of route of administration.

### Limitations

The model is better suited to study only cognitive deficits but not for studying the pathology of AD.

***18. Phenytoin induced memory deficits:*** Phenytoin is a commonly used anti-epileptic drug, effective against all types of partial and tonic–clonic seizures. The treatment with this drug is known to be associated with cognitive decline [59] along with decrease in blood folic acid levels [60]. Administration of phenytoin to rodents caused alterations in the levels of neurotransmitters such as 5-HT along with changes in the cholinesterase activity [61].

### Advantages

This model is beneficial to explore the cognitive deficits associated with dementia and epilepsy.

This model is beneficial for screening memory enhancing agents.

### Limitations

This is a non-reliable model for exploring the pathology of AD.

***19. D-Galactose induced cognitive impairment:*** D-galactose (D-gal) is a natural reducing sugar that is mainly derived from lactose in the milk. Studies have showed that an excess of D-gal leads to abnormal metabolism due to the reduction in Na^+^, K^+^, ATPase activity, excessive oxidative stress due to enhanced lipid peroxidation and downregulation of the superoxide dismutase activity [66], neuronal damage due to advanced glycation [62] and neuroinflammation [63], [64], [65] and increased senile plaques formation [66], tau phosphorylation [68] leading to cognitive impairment in rodents [67]. It is known to cause alteration in hippocampal gene expressions [64].

### Advantages

Chronic D-gal administration has been the widely used animal model to investigate aging brain and AD pathology.

All the pathological hallmarks were observed in this model.

Safe and reliable model.

### Limitations

Insulin resistance has been observed after D-galactose treatment, that could lead to diabetes.

***20. (MK-801) Dizocilpine-induced model of AD****:* MK-801 is an NMDA antagonist with anaesthetic and anticonvulsant properties. This drug induces oxidative stress in the prefrontal cortex. MK-801 administration leads to neurodegeneration in areas responsible for cognition, hence memory deficits [69,70]. It inhibits caspase activation [71] and blockade of glutamate leads to elevated Ca^2+^. This elevation of calcium leads to neurotoxicity [72], due to activation of proteases, lipases, and nitric oxide synthases, thereby increasing free radical formation. It also increases glucose metabolism and induces heat shock protein and necrotic cell death in neurons [70,71]. Glutamate transmission has a significant role in behavioural systems such as motor activity, learning, and cognition [73,74]. MK-801 also alters dopamine levels in the prefrontal cortex, resulting in motor dysfunction [73].

### Advantages

Cognitive deficits can be studied using this model.

Behavioural evaluation and screening of drugs for cognitive dysfunction can be studied.

### Limitations

Animals exhibit schizophrenia-like behaviour.

This animal model failed to replicate the pathological hallmarks of AD.

***21. Diazepam model of cognitive dysfunction:*** Diazepam belongs to the class of sedative and hypnotics. According to the research, benzodiazepines can cause amnesia by inhibiting long-term potentiation, a neuronal process that underlies memory and learning. Memory loss has been linked to the use of certain benzodiazepines. It has been demonstrated that benzodiazepine receptor agonists like diazepam (0.5 to 3 mg/kg i.p.) given 30 minutes before acquisition trials cause anterograde amnesia [15,75]. In addition, other benzodiazepines such as the lorazepam (0.06-0.5mg/kg), alprazolam (0.5-0.75 mg/kg), triazolam (0.05- 0.3 mg/kg) have been reported to produce anterograde and retrograde amnesia in rodent models [76,77].

### Advantages

Cognitive deficits can be studied using this model and memory enhancing agents can be screened.

### Limitations

This animal model failed to replicate the pathological hallmarks of AD.

Diazepam could cause CNS depression.

Many animal models are available for Alzheimer's disease. Each model has its own benefits and limitations. But it is important to note that no animal model completely replicates the whole pathology of human AD. However, the currently available animal models are useful in understanding and solving the key problems of AD neurobiology. Some key points must be considered while selecting an animal model, such as the expression of major hallmarks, i.e., amyloid plaques and neurofibrillary tangles.

Construct validity: It means how faithfully the disease pathogenesis is being presented in the selected animal species.

Face validity: Face validity is attained when there is a phenomenological resemblance between the model and the clinical condition. The model bears a resemblance to the condition or specific aspects.

Predictive validity: The predictive validity of an animal model is achieved if the expectations produced by the model can be authenticated in the clinical condition being modelled. They can also be used to find and study drugs that precipitate or aggravate a clinical condition. Predictive models are frequently used to screen new therapeutic targets.

Validation of animal models: Validate the model thoroughly and scientifically, such as assessment of housing and rearing conditions, age, and finally validate the model across different rodent strains.

Choice of animal species: The species differences among rodents (including a variant with more genetic similarity to humans) and the sex differences (females may have oestrogen protective action) are to be considered.

Inclusion of positive control: Many disease models include positive controls in the study. Exceptionally, in Alzheimer's disease, it is not possible. As the pathogenesis is multifactorial, the drugs currently being used for AD are cholinesterase inhibitors and memantine, which are useful only for treating the symptoms of Alzheimer's disease. AD pathogenesis includes tau hyperphosphorylation, amyloid deposition, increased oxidative stress and neuroinflammation, and alterations in various neurotransmitters along with memory impairment. So, it would not be possible for the currently available AD drugs to act as a standard drug/positive control.

The expression of various core markers/factors such as oxidative stress markers, neuroinflammatory markers, and changes in neurotransmitter levels should be considered. It is hard to decipher the other downstream pathological targets in detail. But the expression of Amyloid Precursor Protein (APP), Presenilin 1, and other cleaved by-products of an APP like AICD may also exert toxicity.

The most important point to be considered is cognitive dysfunction. Most models show memory impairment to some extent, but the timing and the type (stress-related/ age-related) must be cautiously considered in animal studies. For example, memory impairment arises at a different stage of pathogenesis in transgenic rodent models in contrast to humans. Memory impairment occurring at or before the onset of plaque development in rodents should be noted.

Behavioural assessment: Cognitive testing in humans includes learning, speech, social cognition, perception, short-term and long-term memory, and these are determined according to a series of tests like the Cambridge Neuropsychological Test Automated Battery (CANTAB) [78]. However, the distinct cognitive damage is mainly dependent on the disease stage and existing comorbidities. In animal models, this differentiation in disease severity is not reflected and mostly single outcome measures are used. Moreover, the cognitive validation of maze tests is still a topic of dispute.

Poor internal validity: The results of the study might also be influenced by variations in how the test is carried out. For example, in the behavioural assessment by Morris water maze test, differences in the size of the pool, water temperature and days of training, which are being used most frequently.

Animal models have their apparent advantages, such as testing for *in vivo* toxicity studies for new therapeutics, cognitive and behavioural testing, and many more. Selection and validation of the model is the core factor to be considered for any pre-clinical study to get better output.

Poor study design: Animal studies are to be designed, executed, and reported properly using PREPARE, ARRIVE, guidelines. The compliance must be verified by the head/chief.

## 3.Conclusion

AD remains as a disease with a complex pathogenesis having multiple aetiologies, so designing therapeutic strategies for a disease having potential cross-talks remains difficult. Animal models of AD continue to play an important role in pre-clinical research, mainly for the identification of new therapeutics. Drug development for Alzheimer's disease has generally failed, with a failure rate of more than 99%. These failures were caused, in part, by the fact that data acquired from animal models were not being translated to the clinical level. Given this circumstance, it has been questioned whether using animal models to study AD is even worthwhile, particularly when evaluating the effectiveness of novel therapeutics. Low internal validity has primarily been the main reason for poor translation of animal model. Animal studies are frequently poorly designed, underpowered, and poorly standardized (e.g., non-randomized, non-blinded) and it is not possible to replicate all pathological events in a single animal model.

This article presents selection an animal model for AD that is appropriate, such as one that has been used previously or is often utilized, may not be the best choice. We have tried to cover the regimen for induction of AD and major guidelines to be noted for the selection of the animal model. We presumed complete face validity of the animal models, i.e. are the same symptoms present in the model and the human disease, since we only included studies where memory deficits was observed and evaluated. Based on this, the most commonly employed chemically induced models were STZ and β-amyloid models, while d-galactose, scopolamine, aluminium-induced models are being used due to their non-invasive nature, reproducibility, and compatibility. Of the many accessible animal models, transgenic models have proven useful in studying key disease mechanisms implicated in AD. It is important to adjust and revise the models to represent clinical conditions. Yet, regardless of many constraints, animal models have provided valuable knowledge concerning the pathogenesis of AD.

## Ethics statements

Not Applicable.

## CRediT authorship contribution statement

**Deepthi Rapaka:** Conceptualization, Methodology, Writing – original draft. **Paul C. Adiukwu:** Visualization, Supervision. **Veera Raghavulu Bitra:** Writing – review & editing.

## Declaration of interests

The authors declare that they have no known competing financial interests or personal relationships that could have appeared to influence the work reported in this paper.

The authors declare the following financial interests/personal relationships which may be considered as potential competing interests:

*Authors declare no conflict of interest.*

## Data Availability

No data was used for the research described in the article.
